# Study of Correlations between Cognitive Performance, Age, and Education in the MAPS-T Screening Test

**DOI:** 10.1192/j.eurpsy.2025.1843

**Published:** 2025-08-26

**Authors:** M. B. Martins, J. Martini, M. R. Zibetti, J. J. Schneider, C. M. Trentini

**Affiliations:** 1 Assessment and Measurement in Psychology, Federal University of Rio Grande do Sul, Porto Alegre; 2 Psychopathological States and Psychotherapeutic Approaches, University of Vale do Rio dos Sinos, São Leopoldo, Brazil

## Abstract

**Introduction:**

The MAPS-T is a screening instrument currently under development in Brazil, designed for patients over 50 years of age and administered in a computerized format, either online or with assistance. Its purpose is to assess memory binding abilities, which involve integrating complex elements into unified representations, crucial for both short- and long-term memory. Conjunctive binding in short-term memory is responsible for the temporary retention of associations or combinations of features, such as color and shape. Screening instruments like the MAPS-T aim to be low-cost, quick, and non-invasive tools that provide indicators of potential clinical conditions.

**Objectives:**

To investigate the relationship between performance on memory tasks involving binding and the variables age and educational level.

**Methods:**

A total of 33 individuals aged between 50 and 78 years (M=62.09; SD=6.67) with 6 to 35 years of education (M=19.88; SD=5.63) were evaluated. Participants with reported neurological/psychiatric conditions or uncorrected sensory impairments were excluded. Data collection was conducted on a computer by a trained administrator in sessions lasting 15 minutes. The memory binding task required the recognition of a nameable figure and the color and geometric shape surrounding it. Data were analyzed using Spearman’s correlation.

**Results:**

Spearman’s correlation coefficients indicated that age did not show a significant correlation with total recognition, binding score, or dichotomous score (p > 0.05), suggesting that this variable does not have a relevant association with performance in these scores. In contrast, education demonstrated a moderate and significant correlation with total recognition, binding score, and dichotomous score (p < 0.05), suggesting that more years of education are associated with better performance in these areas.

**Descriptive Statistics**

**Table:** 

	N	Minimum	Maximum	Mean	Std. Deviation
age education_level_years Valid N (listwise)	33 33 32	50,00 6	78,00 35	62,0909 19,88	6,67254 5,628

**Table:** 

		Age	education_level_years
MAPS-T - total_recognition_phase_2	Correlation Coefficient	,194	,431*
	Sig. (2-tailed)	,280	,012
	N	33	33
MAPS-T - binding_score	Correlation Coefficient	,198	,383*
	Sig. (2-tailed)	,268	,028
	N	33	33
MAPS-T - dichotomous_score	Correlation Coefficient	,181	,406*
	Sig. (2-tailed)	,313	,019
	N	33	33

**Conclusions:**

Education showed a positive and consistent association with performance across all test measures (total recognition, binding score, and dichotomous score). Age, in turn, did not show a significant correlation with these variables, indicating that, in this sample, education is a more important factor than age in explaining performance on the MAPS-T scores, particularly in the binding stage.

**Disclosure of Interest:**

None Declared

